# On flavourful Easter eggs for New Physics hunger and lepton flavour universality violation

**DOI:** 10.1140/epjc/s10052-017-5270-2

**Published:** 2017-10-17

**Authors:** Marco Ciuchini, António M. Coutinho, Marco Fedele, Enrico Franco, Ayan Paul, Luca Silvestrini, Mauro Valli

**Affiliations:** 1 INFN, Sezione di Roma Tre, Via della Vasca Navale 84, 00146 Rome, Italy; 2 Dipartimento di Matematica e Fisica, Università di Roma Tre, Via della Vasca Navale 84, 00146 Rome, Italy; 3 Dipartimento di Fisica, Università di Roma “La Sapienza”, P.le A. Moro 2, 00185 Rome, Italy; 4 INFN, Sezione di Roma, P.le A. Moro 2, 00185 Rome, Italy

## Abstract

Within the standard approach of effective field theory of weak interactions for $\varDelta B = 1$ transitions, we look for possibly unexpected subtle New Physics effects, here dubbed “flavourful Easter eggs”. We perform a Bayesian global fit using the publicly available HEPfit package, taking into account state-of-the-art experimental information concerning these processes, including the suggestive measurements from LHCb of $R_{K}$ and $R_{K^{*}}$, the latter available only very recently. We parametrise New Physics contributions to $b \rightarrow s$ transitions in terms of shifts of Wilson coefficients of the electromagnetic dipole and semileptonic operators, assuming CP-conserving effects, but allowing in general for violation of lepton flavour universality. We show how optimistic/conservative hadronic estimates can impact quantitatively the size of New Physics extracted from the fit. With a conservative approach to hadronic uncertainties we find nonzero New Physics contributions to Wilson coefficients at the level of $\sim 3\sigma$, depending on the model chosen. Furthermore, given the interplay between hadronic contributions and New Physics effects in the leptonic vector current, a scenario with nonstandard leptonic axial currents is comparable to the more widely advocated one with New Physics in the leptonic vector current.

## Introduction

Easter eggs nowadays also refer to inside jokes and/or secret messages usually hidden e.g. in computer gaming and hi-tech software. In this work, we take advantage of this terminology to motivate the search for New Physics Beyond the Standard Model in the radiative and in the (semi)leptonic channels of rare *B* meson decays.

In the decades that have followed the original formulation of flavour mixing [1], the flavour structure of the SM has been experimentally tested and well established. The tremendous progress of the experimental facilities has probed the flavour of the SM to an exquisite level of precision [2], along with the substantial effort on the part of the theoretical community to go well beyond leading order computations [3]. From this perspective of “precision tests”, radiative and (semi)leptonic $\varDelta B = 1$ processes, related at the partonic level to $b \rightarrow s \gamma , s \ell \ell$ transitions, occupy a special place in probing the SM and its possible extensions in terms of New Physics (NP) models [4, 5].

Firstly, these rare *B* meson decays belong to the class of flavour-changing neutral current (FCNC) processes, which are well known to be sensitive probes of Physics Beyond the Standard Model (BSM): in fact – within the SM – the flavour structure of the theory allows FCNC to arise only at loop level, as a consequence of the GIM mechanism [6]. This allows for significant room for heavy new degrees of freedom to sizeably contribute to these rare processes.

Secondly, from the experimental side, the study of rare *B* meson decays offers us some of the most precise measurements amongst the $| \varDelta F | = 1$ processes. For instance, the measurement of the inclusive branching fraction of $B \rightarrow X_{s} \gamma$ is currently performed with a relative uncertainty of a few percent [7–9], while the study of an exclusive mode such as $B \rightarrow K^{*} \ell \ell$ allows for a detailed analysis of the angular distribution of the four final state particles, yielding rich experimental information in terms of angular functions of the dilepton invariant mass, with full kinematic coverage of the latter [10] and – starting from Ref. [11] – also with available experimental correlations among the angular observables.

In *B* Physics, the recent years have been characterised by the emergence of a striking pattern of anomalies in multiple independent studies of some of these rare $b \rightarrow s$ transitions [12]. Of particular importance, the measurement of the $P_{5}'$ angular observable [13–16] stands out from all the other ones related to the angular distribution of $B \rightarrow K^{*} \mu \mu \,$; first realised by the LHCb collaboration [17, 18] and later on also by the Belle collaboration [19], the experimental analysis of $P_{5}'$ in the large recoil region of the decay points to a deviation of about $3\sigma$ with respect to the SM prediction presented in Ref. [20]. The latter, however, suffers from possible hadronic uncertainties which are sometimes even hard to guesstimate [21–24], and this observation has been at the origin of a quite vivid debate in the recent literature about the size of (possibly) known and (yet) unknown QCD power corrections to the amplitude of this process in the infinite mass limit [25–28]. To corroborate even more the cumbersome picture of the “$P_{5}'$ anomaly”, two new independent measurements of this angular observable (among others) have been recently released by the ATLAS [29] and CMS [30] collaborations, showing, respectively, an appreciable increase and reduction of the tension between data and the SM prediction in Ref. [20], as reported by these experiments.

For the sake of completeness, one should also remark that other smaller tensions have been around, concerning the measurement of differential branching fractions of $B \rightarrow K \mu \mu \,$ [31, 32] and $B_{s} \rightarrow \phi \mu \mu$ [33]. It is worth noting that, while for the latter mode an explanation in terms of hadronic physics may easily be conceivable, the theoretical computation of the former seems to be under control [34].

Quite surprisingly, a possible smoking gun for NP in rare *B* meson decays already came out in 2014, when the LHCb collaboration presented for the first time the measurement of the ratio of branching fractions [35]:

1 $$\begin{aligned} R_{{K}_{[1,6]}}\equiv & {} \frac{Br(B^{+} \rightarrow K^{+} \mu ^{+} \mu ^{-})}{Br(B^{+} \rightarrow K^{+} e^{+} e^{-})} \nonumber \\= & {} 0.745 ^{+ 0.090}_{-0.074}\pm 0.036, \end{aligned}$$

where the subscript refers to the dilepton mass (denoted hereafter $q^{2}$) range going from 1 to 6 GeV$^{2}$. This experimental value shows a deviation of about $2.6\sigma$ with respect to the standard theoretical prediction. Indeed, the SM value of $R_{K}$ in the bin provided by the LHCb collaboration is expected to be equal to unity beyond the percent level of accuracy [36, 37]. In fact, contrary to observables such as $P_{5}'$, it is important to stress that $R_{{K}}$ may be, in general, regarded as insensitive to QCD effects [36]. From the model building point of view, $R_K$ can certainly be considered as quite informative, hinting at a UV completion of the SM where lepton flavour universality violation (LFUV) takes place in the flavour-violating couplings of new heavy degrees of freedom, e.g. leptoquarks and/or $Z'$ gauge bosons [38–67]. Most importantly, the tantalising correlation of this signature of LFUV with the $P_{5}'$ anomaly, suggested by several global analyses [4, 68–72] has triggered different proposals of measurements of such effect in the angular analysis of the $K^{*} \ell \ell$ channel [73, 74]. Interestingly enough, an analysis from the Belle collaboration aiming at separating the leptonic flavours in $B \rightarrow K^{*} \ell \ell$ [75] shows a consistent $\sim 2.6\sigma$ deviation from the SM prediction reported in Ref. [20] in the dimuon leptonic final state only. This is compatible with previous experimental findings related only to the mode with muonic final states.

Sitting on similar theoretical grounds to $R_{K}$, another intriguing ratio of *B* decay branching fractions can be measured in the $K^{*}$ channel:

2 $$\begin{aligned}&R_{{K^{*}}_{[0.045,1.1]}} \,\equiv \, \frac{Br(B \rightarrow K^{*} \mu ^{+} \mu ^{-})}{Br(B \rightarrow K^{*} e^{+} e^{-})}\nonumber \\&~~~~~~~~~~~~~~~~ = 0.660 ^{+ 0.110}_{-0.070}\pm 0.024 ,\end{aligned}$$

3 $$\begin{aligned}&R_{{K^{*}}_{[1.1,6]}} = \ 0.685 ^{+ 0.113}_{-0.069}\pm 0.047 . \end{aligned}$$

These measurements for the low-$q^2$ bin and the central-$q^2$ one have just been presented by the LHCb collaboration [76], pointing again to a discrepancy of about $2\sigma$ with respect to the expected SM prediction – again equal to 1 to a very good accuracy for the central-$q^2$ bin and close to 0.9 for the low-$q^2$ one – and yielding more than a $3\sigma$ deviation when naively combined with the measurement of $R_K$. Note that with higher degree of braveness (or, depending on the taste of the reader, of unconsciousness), the disagreement of the SM with precision *B* physics may reach the exciting level of $\gtrsim 5\sigma$ when one naively combines together the single significances coming from $R_{K,K^{*}}$ ratios, $P_{5}'$ measurements and the minor deviations observed in the other exclusive branching fractions.

**Fig. 1:**
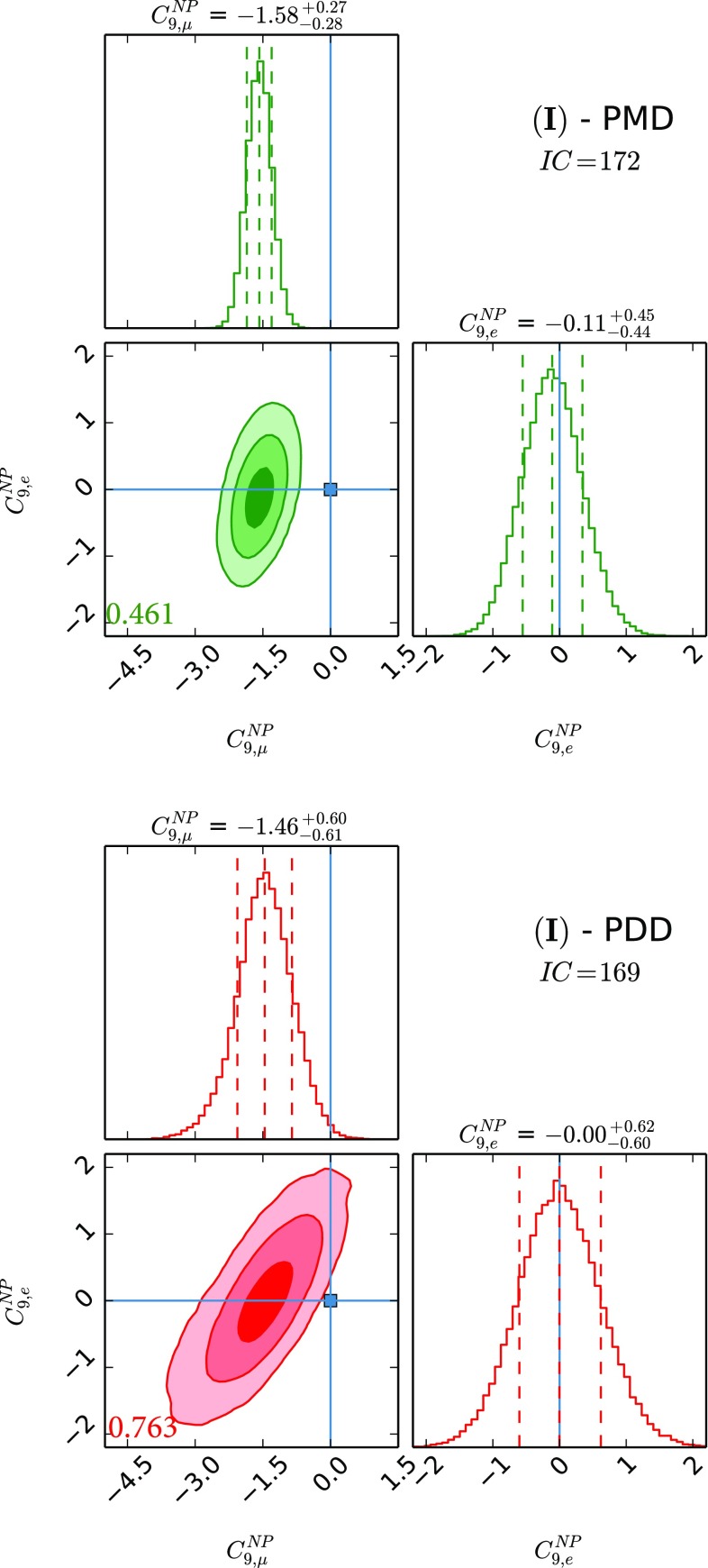
The two NP parameter fit using $C^\mathrm{{NP}}_{9,\mu }$ and $C^\mathrm{{NP}}_{9,e}$. Here and in the following, the left green panel shows the results for the PMD approach and the right red panel shows that for the PDD one. In the 1D distributions we show the 16th, 50th and 84th percentile marked with the dashed lines. In the correlation plots we show the 1, 2 and $3\sigma$ contours in decreasing degrees of transparency. The blue square and lines identify the values of the NP WCs in the SM limit. The numbers at the bottom left corner of the 2D plots refer to the correlation. We also report *IC* values for the two approaches (see Eq. (9)). Preferred models are expected to give smaller *IC* values

**Fig. 2:**
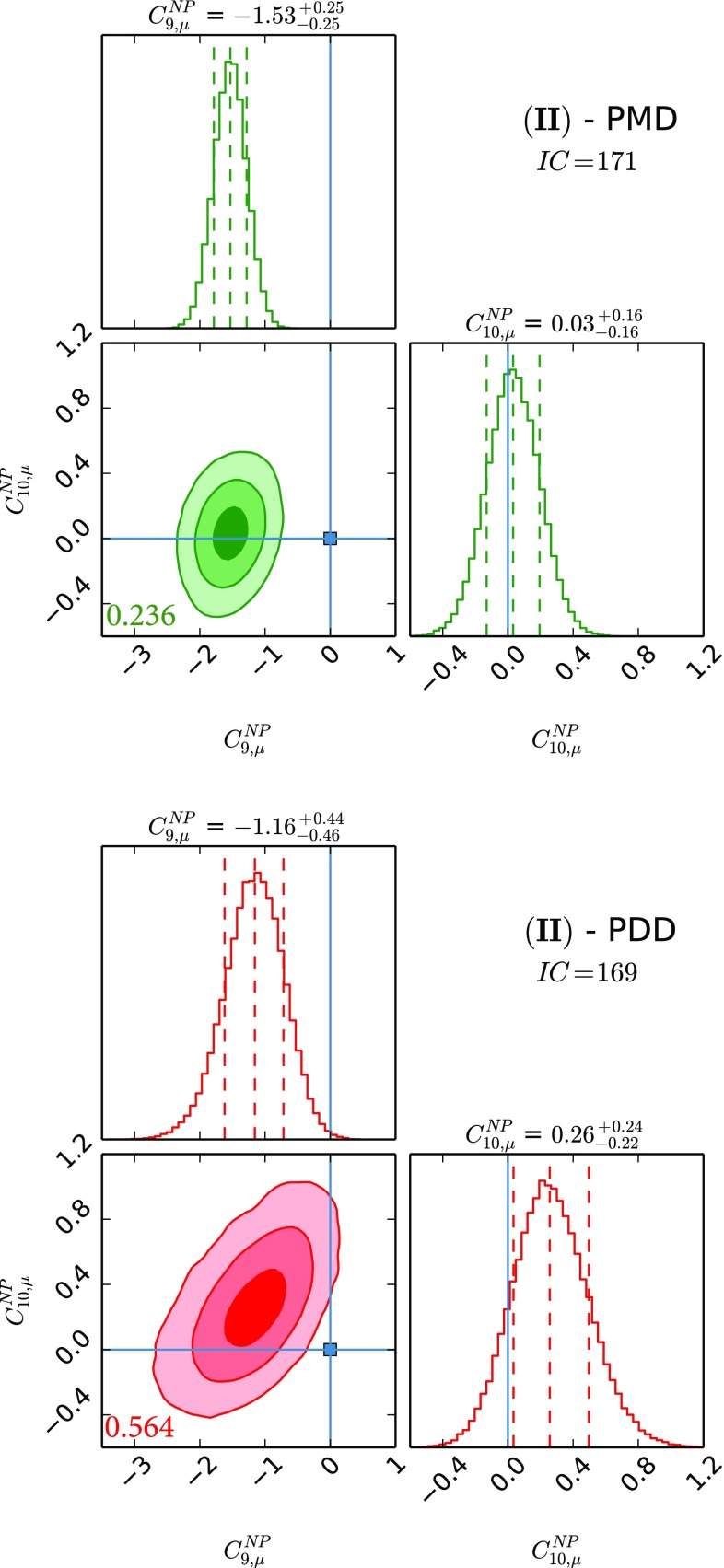
The two NP parameter fit using $C^\mathrm{{NP}}_{9,\mu }$ and $C^\mathrm{{NP}}_{10,\mu }$. See caption of Fig. 1 for the colour coding and further details

**Fig. 3:**
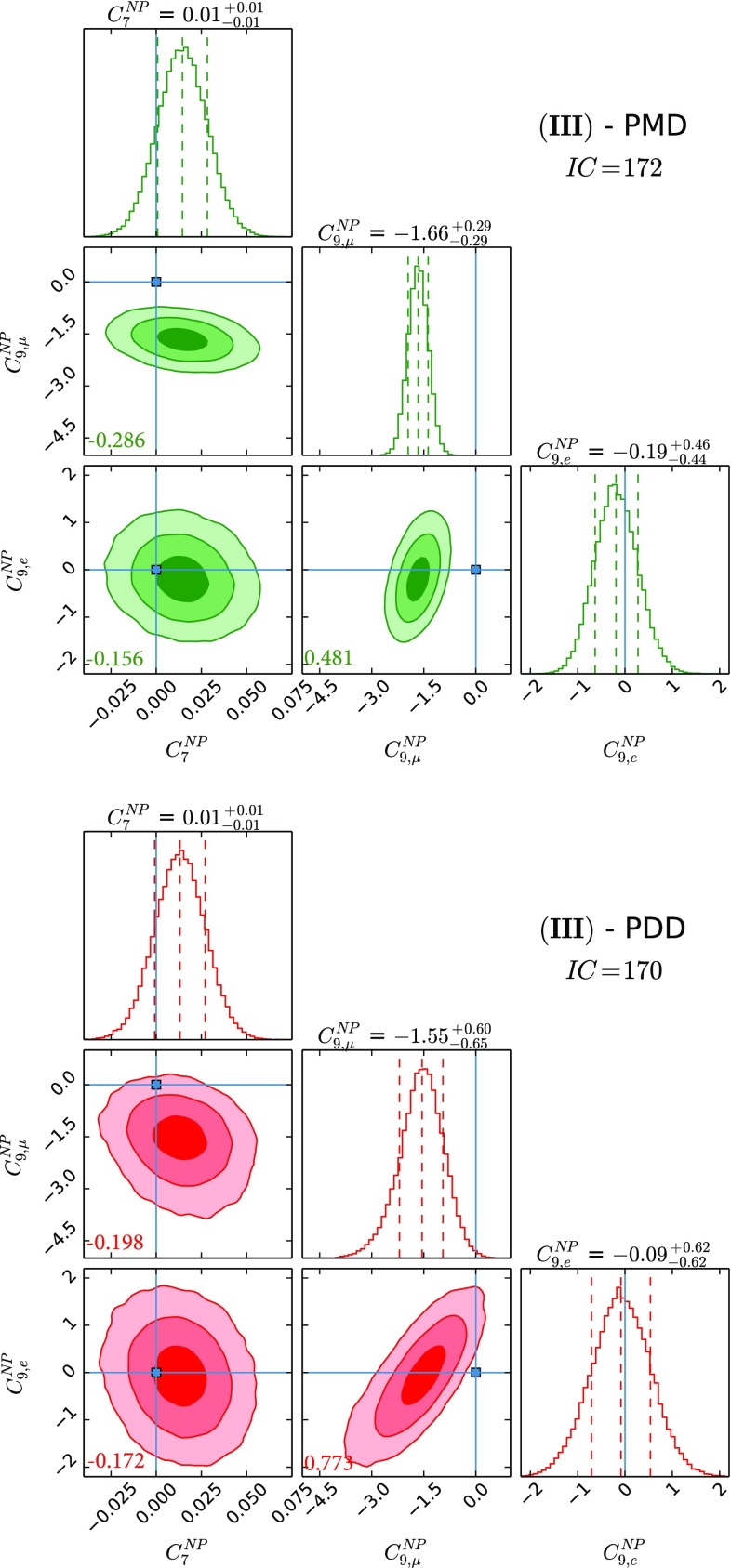
The three NP parameter fit using $C^\mathrm{{NP}}_{7}$, $C^\mathrm{{NP}}_{9,\mu }$ and $C^\mathrm{{NP}}_{9,e}$. See caption of Fig. 1 for the colour coding and further details

**Fig. 4:**
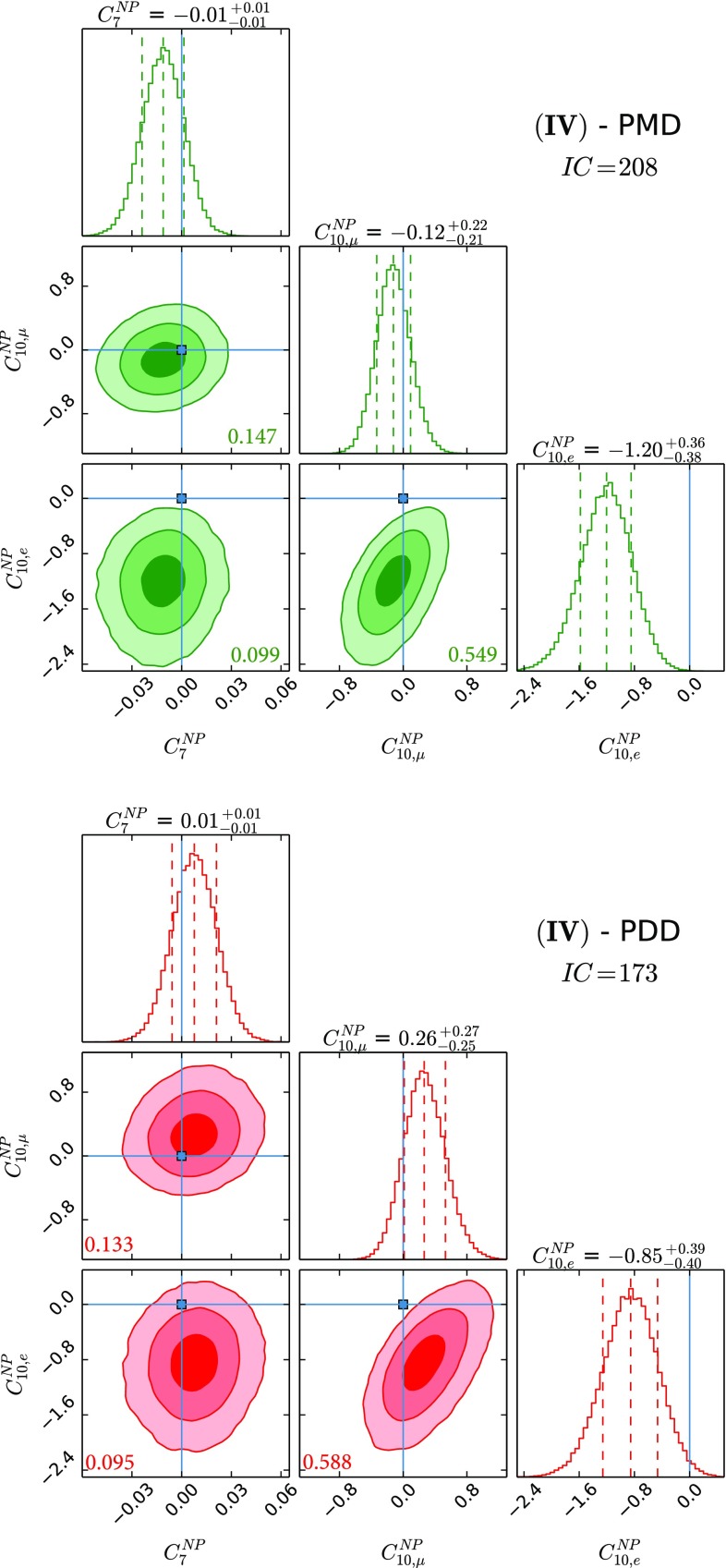
The three NP parameter fit using $C^\mathrm{{NP}}_{7}$, $C^\mathrm{{NP}}_{10,\mu }$ and $C^\mathrm{{NP}}_{10,e}$. See caption of Fig. 1 for the colour coding and further details

**Fig. 5:**
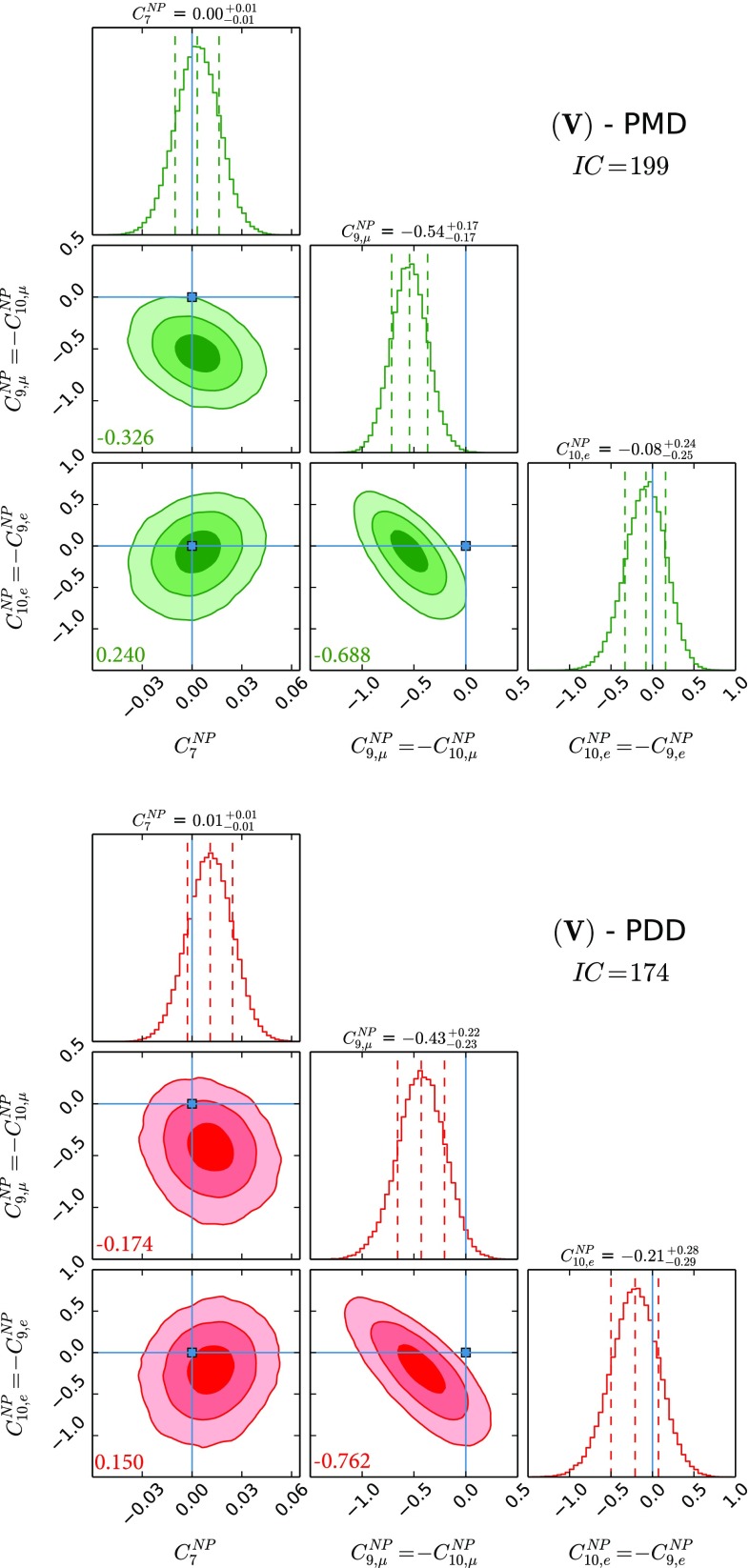
The three NP parameter fit using $C^\mathrm{{NP}}_{7}$, $C^\mathrm{{NP}}_{9,\mu }$, $C^\mathrm{{NP}}_{9,e}$ and $C^\mathrm{{NP}}_{10,\mu ,e}=-C^\mathrm{{NP}}_{9,\mu ,e}$. See caption of Fig. 1 for the colour coding and further details

**Fig. 6:**
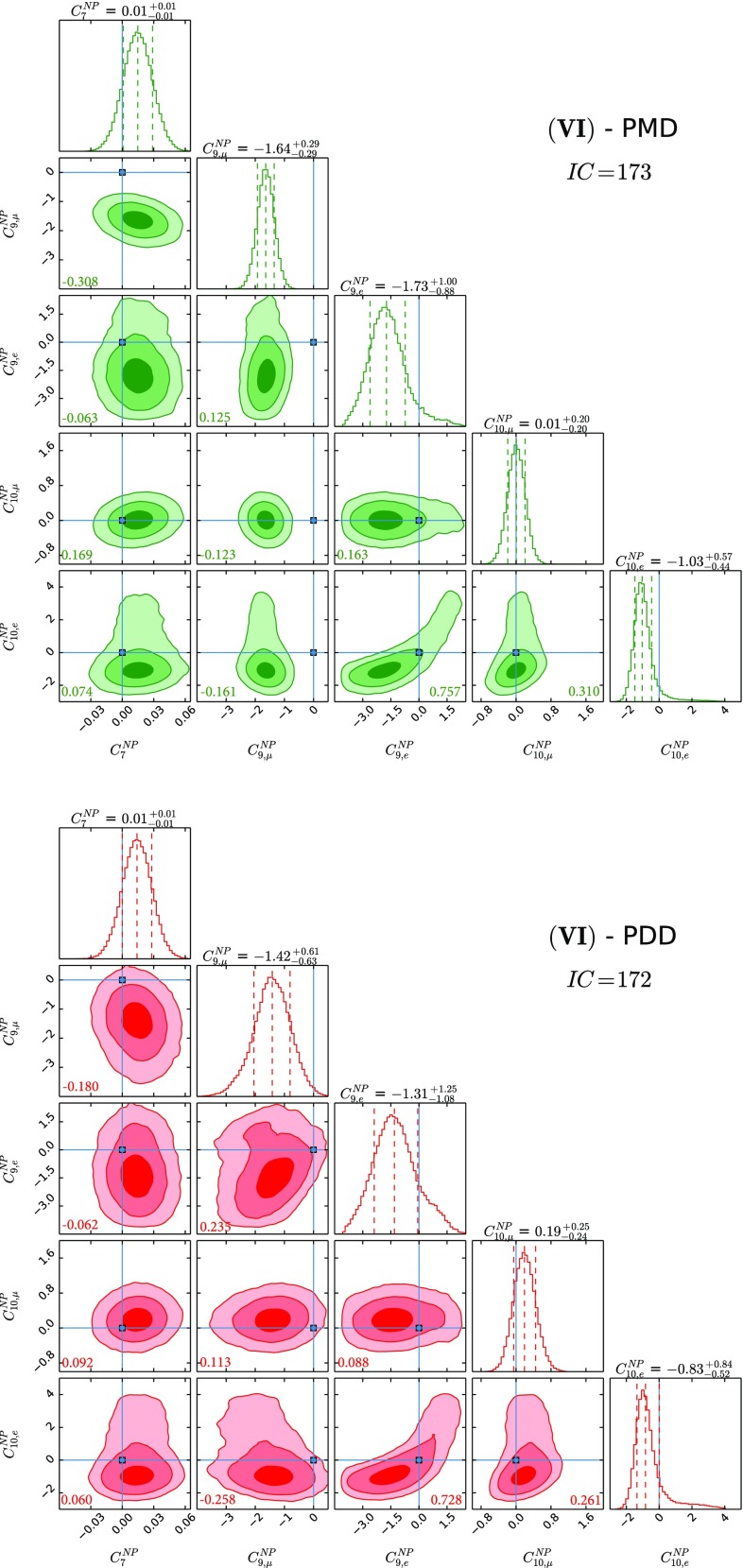
The five NP parameter fit using $C^\mathrm{{NP}}_{7}$, $C^\mathrm{{NP}}_{9,\mu }$, $C^\mathrm{{NP}}_{9,e}$, $C^\mathrm{{NP}}_{10,\mu }$ and $C^\mathrm{{NP}}_{10,e}$. See caption of Fig. 1 for the colour coding and further details

Given the excitement of these days for all the above hints of a possible NP discovery in rare *B* meson decays, in this work we take our first steps towards a positive attitude in the search of a definite BSM pattern aimed at addressing these *B* anomalies. We perform our study in a model-independent fashion, within the framework of effective field theories for weak interactions [77–79]. In particular, in Sect. 2 we define the setup characterising the whole global analysis, presenting six different benchmark scenarios for NP, together with a discussion as regards two different approaches in the estimate of the hadronic uncertainties that can affect quantitatively our final results. In Sect. 3, we list all the experimental measurements we use to construct the likelihood in our fit, and we discuss in detail our most important findings. The latter are effectively depicted in Figs. 1, 2, 3, 4, 5, 6, and collected in Tables 2, 3, 4, 5 in Appendix A. In Sect. 4 we summarise our conclusions.

## Theoretical framework of the analysis

In this section we present the effective field theory framework at the basis of this work and introduce the benchmark scenarios we focus on for our study of NP effects in rare *B* decays. We then illustrate the two distinct broad classes of assumptions that characterise our global analysis: the case where we take an optimistic attitude towards the estimate of hadronic uncertainty plaguing the amplitude of both $B \rightarrow K^{*} \ell \ell / \gamma$ and $B_s \rightarrow \phi \ell \ell / \gamma$ channels, and a second one where we aim at providing a more conservative approach. All the results in Sect. 3.2 will be classified under these two different setups.

### New Physics benchmarks for $\varDelta B = 1$

Integrating out the heavy degrees of freedom, the resulting effective Hamiltonian of weak interactions for $b \rightarrow s \gamma , s \ell \ell$ transitions involves the following set of dimension six operators within the SM [80]:

4 $$\begin{aligned}&Q^p_1 = (\bar{s}_L\gamma _{\mu }T^a p_L)(\bar{p}_L\gamma ^{\mu }T^ab_L),\nonumber \\&Q^p_2 = (\bar{s}_L\gamma _{\mu } p_L)(\bar{p}_L\gamma ^{\mu }b_L), \nonumber \\&P_3 = (\bar{s}_L\gamma _{\mu }b_L)\sum \phantom {} _q(\bar{q}\gamma ^{\mu }q), \nonumber \\&P_4 = (\bar{s}_L\gamma _{\mu }T^ab_L)\sum \phantom {} _q(\bar{q}\gamma ^{\mu }T^aq),\nonumber \\&P_5 = (\bar{s}_L\gamma _{\mu 1}\gamma _{\mu 2}\gamma _{\mu 3}b_L)\sum \phantom {} _q(\bar{q}\gamma ^{\mu 1}\gamma ^{\mu 2}\gamma ^{\mu 3}q),\nonumber \\&P_6 = (\bar{s}_L\gamma _{\mu 1}\gamma _{\mu 2}\gamma _{\mu 3}T^ab_L)\sum \phantom {} _q(\bar{q}\gamma ^{\mu 1}\gamma ^{\mu 2}\gamma ^{\mu 3}T^aq), \\&Q_{8g} = \frac{g_s}{16\pi ^2}m_b\bar{s}_L\sigma _{\mu \nu }G^{\mu \nu }b_R,\nonumber \\&Q_{7\gamma } = \frac{e}{16\pi ^2}m_b\bar{s}_L\sigma _{\mu \nu }F^{\mu \nu }b_R\,, \nonumber \\&Q_{9V} = \frac{\alpha _{e}}{4\pi }(\bar{s}_L\gamma _{\mu }b_L)(\bar{\ell }\gamma ^{\mu }\ell ), \nonumber \\&Q_{10A} = \frac{\alpha _{e}}{4\pi }(\bar{s}_L\gamma _{\mu }b_L)(\bar{\ell }\gamma ^{\mu }\gamma ^5\ell ),\nonumber \end{aligned}$$

where $\ell = e , \mu$, $p = u,c$ and we have neglected the chirally suppressed SM dipoles. The $\varDelta B = 1$ effective Hamiltonian can be cast in full generality in the form of a combination of two distinct parts:

5 $$\begin{aligned} \mathcal {H}_\mathrm {eff}^{\varDelta B = 1} = \mathcal {H}_\mathrm {eff}^\mathrm {had} + \mathcal {H}_\mathrm {eff}^\mathrm {sl+\gamma }, \end{aligned}$$

where, within the SM, the hadronic term involves the first seven operators in Eq. (5):

6 $$\begin{aligned} \mathcal {H}_\mathrm {eff}^\mathrm {had}&= \frac{4G_F}{\sqrt{2}} \Bigg [\sum _{p=u,c}\lambda _p\bigg (C_1 Q^{p}_1 + C_2 Q^{p}_2\bigg ) \nonumber \\&\quad -\, \lambda _t \bigg (\sum _{i=3}^{6} C_i P_i + C_{8}Q_{8g} \bigg )\Bigg ], \end{aligned}$$

while the second piece includes the electromagnetic dipole and semileptonic operators:

7 $$\begin{aligned} \mathcal {H}_\mathrm {eff}^\mathrm {sl+\gamma } = - \frac{4G_F}{\sqrt{2}}\lambda _t ( C_7Q_{7\gamma } + C_9Q_{9V} + C_{10}Q_{10A}), \end{aligned}$$

with $$\lambda _{i}$$ corresponding to the CKM combination $$V^{}_{ib} V^{*}_{is} $$ for $$i=u,c,t$$ and where $$C_{i=1,\ldots ,10}$$ are the Wilson coefficients (WCs) encoding the short-distance physics of the theory. All the SM WCs in this work are evolved from the mass scale of the W boson down to $$\mu _{b}=4.8$$ GeV, using state-of-the-art perturbative QCD and QED calculations for the matching conditions [81–83] and the anomalous dimension matrices [83–86] relevant for the processes considered in this analysis.

While a general UV completion of the SM may enter in the effective couplings present in both pieces of Eq. (5), general NP effects in $b \rightarrow s \gamma , s \ell \ell$ can be phenomenologically parametrised as shifts of the Wilson coefficients of the electromagnetic and semileptonic operators at the typical scale of the processes, $\mu _{b}$. In particular, the most general basis for NP effects in radiative and (semi)leptonic *B* decays can be enlarged by the presence of scalar, pseudo-scalar and tensorial semileptonic operators, together with right-handed quark currents as the analogue of $Q_{7\gamma } ,Q_{9V},Q_{10A}$ SM operators [21, 87]. In this work, motivated by previous interesting findings concerning LFUV [69–71] and the measurement of $R_{K}$ and $R_{K^{*}}$, we focus on the contributions of NP appearing as shifts of the SM WCs related to the electromagnetic dipole and semileptonic operators with left-handed quark currents only. A comprehensive analysis with different chiral structures as well as a more general effective theory framework will be presented elsewhere [88]. Furthermore, we restrict ourselves to CP-conserving effects, taking NP WCs to be real.

For NP in semileptonic operators we discriminate between couplings to muon and electron fields both in the axial and vector leptonic currents. We characterise our phenomenological analysis for NP through six different benchmark scenarios, studying the impact of combinations of the following NP WCs:

(I): $C^\mathrm{{NP}}_{9,\mu }$ and $C^\mathrm{{NP}}_{9,e}$ varied in the range $[-4,4]$, i.e. adding to the SM two NP parameters;
(II): $C^\mathrm{{NP}}_{9,\mu }$ and $C^\mathrm{{NP}}_{10,\mu }$ varied in the range $[-4,4]$, adding to the SM again two NP parameters;
(III): $C^\mathrm{{NP}}_{9,\mu }$ and $C^\mathrm{{NP}}_{9,e}$ varied in the range $[-4,4]$, and $C^\mathrm{{NP}}_{7}$ varied in the range $[-0.5,0.5]$, i.e. a scenario with three NP parameters;
(IV): $C^\mathrm{{NP}}_{10,\mu }$ and $C^\mathrm{{NP}}_{10,e}$ varied in the range $[-4,4]$, and $C^\mathrm{{NP}}_{7}$ varied in the range $[-0.5,0.5]$, i.e. adding again to the SM three NP parameters;
(V): $C^\mathrm{{NP}}_{9,\mu } = - C^\mathrm{{NP}}_{10,\mu }$ and $C^\mathrm{{NP}}_{9,e} = - C^\mathrm{{NP}}_{10,e}$ varied in the range $[-4,4]$, and $C^\mathrm{{NP}}_{7}$ varied in the range $[-0.5,0.5]$, i.e. a NP scenario again described by three different parameters.
(VI): $C^\mathrm{{NP}}_{7}$, $C^\mathrm{{NP}}_{9,\mu }$, $C^\mathrm{{NP}}_{9,e}$, $C^\mathrm{{NP}}_{10,\mu }$ and $C^\mathrm{{NP}}_{10,e}$ varied simultaneously in the respective ranges defined above, i.e. a NP scenario described by five different parameters.

We remark that while benchmarks (I) and (II) have been already studied in the literature, none of the other cases has been analysed so far. In particular, NP scenarios (III) and (IV) allow us to study, for the first time, the interesting impact of a NP radiative dipole operator in combination with vector-like and axial-like LFUV effects generated by NP. Most interestingly, scenario (V) allows us to explore the correlation $C_{9}^\mathrm{{NP}}=-C_{10}^\mathrm{{NP}}$, possibly hinting at a $SU(2)_{L}$ preserving BSM theory. As an additional interesting case to explore, we eventually generalise to simultaneously nonvanishing $C^\mathrm{{NP}}_{7}$, $C^\mathrm{{NP}}_{9,\mu }$, $C^\mathrm{{NP}}_{9,e}$, $C^\mathrm{{NP}}_{10,\mu }$ and $C^\mathrm{{NP}}_{10,e}$ in case (VI).

We wish to stress that all of the six benchmarks defined above will be studied for the first time under two different approaches in the estimate of QCD hadronic power corrections, as presented in next section.

### Treatment of the hadronic uncertainties

In our previous work [24, 27, 89], we went into considerable detail on the treatment of hadronic contributions in the angular analysis of $B\rightarrow K^*\ell \ell$. Our approach there was to study how large these contributions can be assuming that the LHCb data on branching fractions and angular distributions of these decay modes could be described within the SM. For that purpose we considered four scenarios for the hadronic contributions, with increasing theoretical input from the phenomenological analysis presented in Ref. [90]. The underlying functional form that we used for the hadronic contribution was given by

8 $$\begin{aligned} h_\lambda (q^2)= & {} \frac{\epsilon ^{*}_\mu (\lambda )}{m_B^2} \int \mathrm{d}^4x e^{iqx} \langle \bar{K}^{*} \vert T\{j^{\mu }_\mathrm {em} (x) \mathcal {H}_\mathrm {eff}^\mathrm {had} (0)\} \vert \bar{B} \rangle \nonumber \\= & {} h_\lambda ^{(0)} + \frac{q^2}{1\,\mathrm {GeV}^2} h_\lambda ^{(1)} + \frac{q^4}{1\, \mathrm {GeV}^4} h_\lambda ^{(2)}, \end{aligned}$$

where we fitted for the complex, helicity dependent, coefficients $h^{(i)}_\lambda$, $(i=0,1,2)$ and $(\lambda =0,+,-)$ using the data and the phenomenological model in [90]. Since $h_0$ enters the decay amplitude with an additional factor of $\sqrt{q^2}$ with respect to $h_\pm$, we drop $h_0^{(2)}$ in our analysis.

In this work we proceed to study the possible existence of NP contributions in semileptonic and radiative $b\rightarrow s$ decays which requires a re-evaluation of the hadronic uncertainties. For the sake of simplicity, to address hadronic contributions we use the same functional parameterisation as given in Eq. (8). However, we limit ourselves to only two hadronic models. The first, corresponding to the most widely used assumption, relies completely on the phenomenological model in [90] below $q^2 < 4m_c^2$. The second is a more conservative approach, where we impose the latter only in the large recoil region at $q^2\le 1$ GeV$^2$ while letting the data drive the hadronic contributions in the higher invariant mass region. We will refer to the first approach as phenomenological model driven (PMD) and the second as phenomenologically and data driven (PDD). In our fit we vary the $h^{i}_\lambda$ parameters over generous ranges. More detailed discussion of these can be found in [24, 27].

In the present analysis we also need to address modes that were not considered in our previous work, namely $B\rightarrow K\ell \ell$, $B_{s}\rightarrow \phi \ell \ell$ and $B_{s}\rightarrow \phi \gamma$. The decay $B\rightarrow K\ell \ell$ has been studied in detail in [34], where the authors show that the hadronic uncertainties are smaller than in $B\rightarrow K^*\ell \ell$. A comparison of the LCSR estimate of the soft gluon contribution and the QCDF estimate of the hard gluon contribution reveals that the soft gluon exchange is subdominant with respect to QCDF hard gluon exchange. Therefore, although in principle the same concerns on the soft gluon contribution we raised for $B \rightarrow K^*$ apply in this case, in practice the overall effect of soft gluons can be reasonably neglected. In our computation we therefore only include hard gluon exchange computed using the QCDF formalism in Ref. [91].

The long distance contributions for $B_s\rightarrow \phi \ell \ell$ and $B_s\rightarrow \phi \gamma$ follow a similar theoretical derivation to those for $B\rightarrow K^*\ell \ell$ and $B\rightarrow K^*\gamma$, respectively, barring the fact that the spectator quark in the former is different from that in the latter. No theoretical estimates of power corrections to the infinite mass limit are available for the $B_s \rightarrow \phi \ell \ell / \gamma$ decays and one has to rely on the ones for the $B\rightarrow K^* \ell \ell / \gamma$ decays to get a handle on the long distance contributions. The spectator quark effects can come through the hard spectator scattering involving matrix elements of $Q_{2}$, $P_6$ and $Q_{8g}$ computable in QCD factorisation [91] which we include in our computation. However, we do not include the sub-leading, and numerically small, QCDF power corrections to spectator scattering involving $Q_{8g}$ [92–94] and contributions to weak spectator scattering involving $Q_{8g}$ beyond QCDF computed in LCSR [95–97]. The effect of the difference in all these spectator contributions is expected to be low firstly because they are numerically small and, secondly, because the effect is proportional to the small flavour *SU*(3) breaking. Different approaches in relating the long distance contributions in the $B\rightarrow K^* \ell \ell / \gamma$ channels to the ones in the $B \rightarrow \phi \ell \ell / \gamma$ channels have been used in the literature [69, 70, 98], which vary in the degree of correlation between the two. While Ref. [70] uses uncorrelated hadronic uncertainties, Refs. [69, 98] have left the two contributions highly correlated noting that the spectator contribution is expected to be numerically small. We take an approach similar to the latter considering the insensitivity of the current data to such effects and use the same value of power corrections in $B \rightarrow K^*$ and $B_s \rightarrow \phi$ amplitudes, even though this choice pertains to a quite oversimplifying optimistic attitude. We leave a more detailed analysis of this assumption by relaxing the correlation between the hadronic contributions in the two modes to a future work [88].

## Bayesian fit of the dipole and semileptonic operators

### Experimental information considered

In this section we discuss the experimental measurements we use in our fit. Note that for the exclusive modes we make use of measurements in the large recoil region only. Our choice harbours on the fact that the QCD long distance effects in the low recoil region are substantially different from the large recoil regime [99–102] and would require a dedicated analysis. For the fit in this study we consider the following experimental information:

- $B \rightarrow K^* \ell \ell$ For the $B \rightarrow K^* \mu \mu$ channel we use the LHCb measurements of CP-averaged angular observables extracted by means of the unbinned maximum likelihood fit, along with the provided correlation matrix [18]. Moreover, we employ the recent results for CP-averaged angular observables from ATLAS [29] and the ones measured by CMS [30, 103].^1^ Finally, we use the CP-averaged optimised angular observables recently measured by Belle [75]^2^. Regarding the differential branching fractions, we use the recently updated measurements from LHCb [104] and the ones from CMS [103]. For the $B \rightarrow K^* e e$ channel we consider the LHCb results from [105] and the Belle results from [75]. $R_{K^*}$ observable is considered according to the recently presented measurements by LHCb [76] in both the low-$q^{2}$ and the central-$q^{2}$ bins; see also Eq. (3). Our theoretical predictions are computed in the helicity basis, whose relevant expressions can be found in [21]; the same framework is employed to study $B \rightarrow K^* \gamma$, $B_s \rightarrow \phi \mu \mu$, $B_s \rightarrow \phi \gamma$ and $B \rightarrow K \ell \ell$ channels. For the latter, we use the full set of form factors extrapolated from the lattice results, along with the provided correlation matrix [106]; for the remaining channels, we use the full set of form factors estimated combining LCSR and lattice results, along with the correlation matrices [107]. For the factorisable and non-factorisable QCD power corrections, we refer to Sect. 2.2.
- $B \rightarrow K^* \gamma$ We include in our analysis the HFAG average for the branching fractions from [2].
- $B_s \rightarrow \phi \mu \mu$ We consider the LHCb CP-averaged angular observables and differential branching fractions measurements, along with the provided correlation matrix [33].
- $B_s \rightarrow \phi \gamma$ We use the LHCb measurement of the branching fraction from [108].
- $B \rightarrow K \ell \ell$ We employ the LHCb measurement of $B \rightarrow K e e$ differential branching fraction and $R_K$ from [35].
- $B \rightarrow X_s \gamma$ We use the HFAG average from [2]. We perform our theoretical computation at NNLO in $\alpha _s$ and NLO in $\alpha _{em}$, following Ref. [109] and the references therein.
- $B_s \rightarrow \mu \mu$ We consider the latest measurement from LHCb [110] and do not consider the measurement from CMS [111], which has the same central value of LHCb, but larger uncertainty. Moreover, we chose not to use results for $B_d \rightarrow \mu \mu$, since there are only upper bounds for this decay channel so far [110, 111]. Our theoretical predictions include NLO EW corrections, as well as an NNLO QCD correction, following the detailed expressions obtained in Ref. [112].

**Table 1:** Parameters used in the analysis. The Gegenbauer parameters and $\lambda _B$ have flat priors with half width reported in the third column. The remaining ones have Gaussian priors. Meson masses, lepton masses, *s*-quark mass and electroweak couplings are fixed at the PDG value [117]

Parameters	Mean value	Uncertainty	References
$\alpha _{s}(M_{Z})$	0.1181	0.0009	[117, 118]
$\mu _{W}$ (GeV)	80.385	−	
$m_{t}$ (GeV)	173.34	0.76	[119]
$m_{c}(m_c)$ (GeV)	1.28	0.02	[120]
$m_{b}(m_b)$ (GeV)	4.17	0.05	[121]
$f_{B_{s}}$ (MeV)	226	5	[122]
$f_{B_{s}}/ f_{B_{d}}$	1.204	0.016	[122]
$\varDelta \varGamma _s/\varGamma _s$	0.129	0.009	[2]
$\lambda$	0.2250	0.0006	[123, 124]
*A*	0.829	0.012	[123, 124]
$\bar{\rho }$	0.132	0.018	[123, 124]
$\bar{\eta }$	0.348	0.012	[123, 124]
$f_{K^*,\vert \vert }$ (MeV)	204	7	[107]
$f_{K^*,\perp }(1\,\,\mathrm {GeV})$ (MeV)	159	6	[107]
$f_{\phi ,\vert \vert }$ (MeV)	233	4	[107]
$f_{\phi ,\perp }(1\,\,\mathrm {GeV})$ (MeV)	191	4	[107]
$\lambda _{B}$ (MeV)	350	150	[125]
$a_{1}(\bar{K}^{*})_{\perp , \, ||}$	0.04	0.03	[126]
$a_{2}(\bar{K}^{*})_{\perp , \, ||}$	0.05	0.1	[127]
$a_{2}(\phi )_{\perp , \, ||}$	0.23	0.08	[128]
$a_{1}(K)$	0.06	0.03	[126]
$$a_{2}(K)$$	0.115	$$ -$$	[129]

### Results of the global fit

In this section we present the main results of our work. We perform this study using HEPfit  [113] relying on its Markov Chain Monte Carlo-based Bayesian analysis framework implemented with BAT [114]. We fit to the data using 16 real free parameters that characterise the non-factorisable power corrections, as was done in [24], along with the necessary set of NP WCs. We assign to the hadronic parameters and the NP WCs flatly distributed priors in the relevant ranges mentioned in Sect. 2. The remaining parameters used in the fit are listed in Table 1. To better compare different scenarios, we use the Information Criterion [115, 116], defined as

9 $$\begin{aligned} IC = -2 \overline{\log L} + 4 \sigma ^2_{\log L}, \end{aligned}$$

where $\overline{\log L}$ is the average of the log-likelihood and $\sigma ^2_{\log L}$ is its variance. The second term in Eq. (9) takes into account the effective number of parameters in the model, allowing for a meaningful comparison of models with different number of parameters. Preferred models are expected to give smaller *IC* values.

The results for NP WCs for the several cases that we study can be found in Figs. 1, 2, 3, 4, 5 and 6, where the *IC* value for each model is also reported, and in Tables 2 and 3 in Appendix A. In Tables 4 and 5, we report the results of the fit for observables of interest. We observe that all cases have comparable *IC* values except cases (IV) and (V), which are disfavoured in the PMD approach while they remain viable in the PDD one. The main difference between the two approaches is that angular observables, in particular $P_5'$, call for NP in $C_{9,\mu }^\mathrm{{NP}}$ in the PMD approach, while they can be accommodated within the SM in the PDD one.

Let us discuss the various cases in more detail. It is important to stress that the evidence of NP in our fit for cases (I)–(V) is always larger than $3\sigma$ for one of the semileptonic NP WCs used in the analysis, given the need of a source of LFUV primarily from $R_{K,K^{*}}$ measurements. In particular, we remark that in the PMD scenarios of cases (I) and (II) we get evidence for NP at more than $5\sigma$. However, looking at the corresponding PDD scenarios, the NP evidence gets significantly reduced, roughly between $3\sigma$ and $4\sigma$. The reduction in the significance comes from the larger hadronic uncertainties in the PDD approach which weaken the constraining power of the angular observables on the NP WCs.

Concerning case (III), we observe very similar findings to the ones obtained for case (I), since the effective coupling for the radiative dipole operator is well constrained, especially from the inclusive $B \rightarrow X_s \gamma$ branching fraction.

Regarding case (IV), in which we vary the three NP parameters $C_{7}^\mathrm{{NP}}, C^\mathrm{{NP}}_{10,\mu }$ and $C^\mathrm{{NP}}_{10,e}$, the model comparison between the PDD and PMD realisation of this NP benchmark is quite informative: NP effects in the dipole operator and in the axial semileptonic currents cannot address at the same time $R_{K,K^{*}}$ ratios and the $P_{5}'$ anomaly in a satisfactory way when we stick to small non-factorisable QCD power corrections; however, this is no longer true when we allow for a more conservative estimate of the hadronic uncertainties. In particular, the tension in the fit coming from the angular analysis of $B \rightarrow K^{*} \mu \mu$ can now be addressed by large QCD effects as those given in Eq. (8), while a $C^\mathrm{{NP}}_{10,e} \ne 0$ at about $3\sigma$ can successfully describe all the observational hints of LFUV showed by current measurements. This interesting possibility of *axial lepton-flavour violating NP* is not found in other global analyses [69–72], as it proceeds from the conservative treatment of hadronic uncertainties we proposed in Ref. [24].

Concerning Tables 4 and 5 of Appendix A, we would like to point out the pattern displayed by the transverse ratios $R_{K^*}^T$ and $R_{\phi }^T$: cases (I)–(III) predict these values to be $\sim 1$ with a small error, while the remaining cases give different predictions with the central value ranging between $\sim 0.7$ and $\sim 0.8$. Therefore, obtaining experimental information on transverse ratios may help in discerning between the different NP scenarios.

We then show results for case (V), in which we vary $C_{7}^\mathrm{{NP}}$, $C_{9,\mu }^\mathrm{{NP}}$, $C_{9,e}^\mathrm{{NP}}$ and correlate the semileptonic vector and axial currents according to $C_{9,\mu }^\mathrm{{NP}}=-C_{10,\mu }^\mathrm{{NP}}$ and $C_{9,e}^\mathrm{{NP}}=-C_{10,e}^\mathrm{{NP}}$. In analogy with case (IV), only within the PDD approach we find for this NP benchmark a fairly good description of data, with $C_{9,\mu }^\mathrm{{NP}} = -C_{10,\mu }^\mathrm{{NP}}$ compatible with zero at $\sim 2\sigma$. Again, we are presented with the case where deviations in angular observables are addressed by large QCD power corrections, while LFUV is driven by semielectronic operators. Looking back at Tables 4 and 5, we note that, for this case, as well as for case (IV) and (VI), both transverse and longitudinal muon over electron ratios in the central-$q^{2}$ bin, namely $R^T_{K,K^{*},\phi } \,$ and $R^L_{K,K^{*},\phi } \,$, are characterised by similar central values.

We close our presentation with an analysis of case (VI) in which we float simultaneously $C_{7}^\mathrm{{NP}}$, $C_{9,\mu }^\mathrm{{NP}}$, $C_{9,e}^\mathrm{{NP}}$, $C_{10,\mu }^\mathrm{{NP}}$, and $C_{10,e}^\mathrm{{NP}}$. As can be seen from Fig. 6, current measurements are informative enough to constrain, at the same time, all the NP WCs both in the PMD and PDD approaches. In particular, within the latter case, a nontrivial interplay among NP effects encoded both in $C_{9,\mu }^\mathrm{{NP}}$ and $C_{10,e }^\mathrm{{NP}}$, together with the hadronic contributions reported in Table 3, produces the weakest hint in favour of NP provided by our global analysis – sitting between $2\sigma$ and $3\sigma$ level – while allowing for a very good description of the entire data set, similar to the other cases. The power corrections we found are larger than those obtained in Ref. [90], but smaller than those required by the SM fit of $B\rightarrow K^*\mu \mu$ [24]. As discussed in detail in Refs. [27, 89], the size obtained for the power corrections is compatible with the naive power counting relative to the leading amplitude. We stress (once again) that a more optimistic attitude towards the estimate of QCD power corrections (PMD approach) leads to the a much stronger claim in favour of NP, at a statistical significance larger than $5\sigma$.

In Tables 2 and 3 we report mean and standard deviation for the NP WCs and absolute values of $h_{\lambda }$ for all the cases considered in the analysis. It is also relevant to observe that, once we switch on NP effects through $C^\mathrm{{NP}}_{9,\mu }$ in order to attempt at simultaneously explaining observables such as $R_{K,K^{*}}$ and $P_{5}'$ in the PDD approach we find values for $|h_\lambda ^{(1,2)}|$ compatible with zero at $\sim 1\sigma$. Conversely, if we set $C^\mathrm{{NP}}_{9,\mu }=0$ then a nonvanishing $|h_{-}^{(2)}|$ is needed to account for the angular observables, as found in Ref. [24], showing that one cannot disentangle hadronic uncertainties and NP in $B\rightarrow K^*\mu \mu$ at present.

## Discussion

In this work, we critically examined several BSM scenarios in order to possibly explain the growing pattern of *B* anomalies, recently enriched by the $R_{K^*}$ measurement performed by the LHCb collaboration [76]. We carried out our analysis in an effective field theory framework, describing the non-factorisable power corrections by means of 16 free parameters in our fit along the lines of Ref. [24].

We performed all our fits using two different hadronic models. The first approach, labelled PMD, relies completely on the phenomenological model from Ref. [90] and corresponds to the more widely used choice in the literature. The second one, named PDD, imposes the result of Ref. [90] only at $q^2\le 1$,^3^ allowing the data to drive the hadronic contributions in the higher invariant mass region.

Regarding the NP contributions, we analyse six different benchmark scenarios, differentiated by distinct choices of NP WCs employed in the fits. Case (I) allows for $C^\mathrm{{NP}}_{9,\mu }$ and $C^\mathrm{{NP}}_{9,e}$, while case (II) considers the scenario with $C^\mathrm{{NP}}_{9,\mu }$ and $C^\mathrm{{NP}}_{10,\mu }$; case (III) studies NP effects coming as $C^\mathrm{{NP}}_{7}$, $C^\mathrm{{NP}}_{9,\mu }$ and $C^\mathrm{{NP}}_{9,e}$, and case (IV) is the same as the latter but with $C^\mathrm{{NP}}_{10}$ instead of $C^\mathrm{{NP}}_{9}$; case (V) studies the possibility described in the third case with $C_{10,\mu }^\mathrm{{NP}} = - C_{9,\mu }^\mathrm{{NP}}$ and $C_{10,e}^\mathrm{{NP}} = - C_{9,e}^\mathrm{{NP}}$ enforced; finally, case (VI) considers the general case with all the five NP WCs being allowed to float independently. Our main results are collected in Figs. 1, 2, 3, 4, 5 and 6 and also reported in Tables 2, 3 and 5.

The comparison of different scenarios using the *IC* shows that all the considered cases are on the same footing except for cases (IV) and (V). These cases are strongly disfavoured in the PMD approach, as there is no $C_{9,\mu }^\mathrm{{NP}}$ in case **(IV)** to account for the deviation in $P_5'$, while $C_{9,\mu }^\mathrm{{NP}}$ is constrained by its correlation with $C_{10,\mu }^\mathrm{{NP}}$ and the measured value of BR$(B_s\rightarrow \mu \mu )$ in case (V).

In fact, from our analysis of radiative and (semi)leptonic *B* decays we identify two classes of viable NP scenarios:

- The widely studied $C^\mathrm{{NP}}_{9,\mu } \ne 0$ scenario: from Figs. 1, 2 and 3, we find a remarkable $\gtrsim 5\sigma$ evidence in favour of $C^\mathrm{{NP}}_{9,\mu } \ne 0$ in the PMD approach. It is indeed nontrivial that a single NP WC can explain all the present anomalies in $b\rightarrow s$ transitions [4, 68–72]. However, in the more conservative PDD approach, the significance of a nonvanishing $C^\mathrm{{NP}}_{9,\mu }$ drops to about $3\sigma$, mainly driven by LFUV.
- An alternative scenario with nonvanishing $C^\mathrm{{NP}}_{10,e}$, which emerges in the presence of large hadronic corrections to the infinite mass limit, namely our PDD approach. To our knowledge, a NP electronic axial current has not been studied in the literature, since it does not provide a satisfactory description of the angular observables within the commonly used PMD approach. We think that the present theoretical status of power correction calculations is not robust enough to discard this interesting NP scenario.

Finally the most general fit we performed, namely case (VI), confirms in the PDD approach that both scenarios above are viable, although a slight preference for $C^\mathrm{{NP}}_{9,\mu } \ne 0$ is found. More data are needed to assess what kind of NP scenario (if the anomalies persist) is realised in Nature.

## Appendix A: Numerical results

In this appendix we present the tables with the most relevant numerical results obtained from our global analysis. Mean and standard deviation for the NP WCs and $h_{\lambda }$ absolute values are reported in Table 2 for the PMD approach and in Table 3 for the PDD one.^4^ In Table 4 we list the results in the PMD approach obtained for the key observables in the six NP scenarios. Analogous results for the PDD approach can be found in Table 5.

**Table 2:** Results from the fit for WCs and hadronic contributions in the PMD approach. See Sect. 2.1 for details of the six NP scenarios

Par.	(I)	(II)	(III)	(IV)	(V)	(VI)
$C_{7}^\mathrm{NP}$	−	−	$0.015 \pm 0.014$	$-0.011 \pm 0.013$	$0.003 \pm 0.013$	$0.015 \pm 0.014$
$C_{9,\mu }^\mathrm{NP}$	$-1.58 \pm 0.28$	$-1.53 \pm 0.25$	$-1.66 \pm 0.29$	−	$-0.54 \pm 0.17$	$-1.64 \pm 0.29$
$C_{9,e}^\mathrm{NP}$	$-0.10 \pm 0.45$	−	$-0.18 \pm 0.46$	−	$0.09 \pm 0.25$	$-1.6 \pm 1.0$
$C_{10,\mu }^\mathrm{NP}$	−	$0.03 \pm 0.16$	−	$-0.12 \pm 0.22$	$0.54 \pm 0.17$	$0.009 \pm 0.200$
$C_{10,e}^\mathrm{NP}$	−	−	−	$-1.22 \pm 0.37$	$-0.09 \pm 0.25$	$-0.91 \pm 0.76$
$|h_0^{(0)}| \cdot 10^4$	$2.1 \pm 1.2$	$2.0 \pm 1.2$	$2.2 \pm 1.3$	$1.8 \pm 1.2$	$1.3 \pm 1.0$	$2.0 \pm 1.3$
$|h_+^{(0)}| \cdot 10^4$	$0.079 \pm 0.067$	$0.079 \pm 0.067$	$0.076 \pm 0.065$	$0.083 \pm 0.069$	$0.086 \pm 0.072$	$0.076 \pm 0.064$
$|h_-^{(0)}| \cdot 10^4$	$0.53 \pm 0.19$	$0.54 \pm 0.19$	$0.52 \pm 0.19$	$0.56 \pm 0.20$	$0.60 \pm 0.21$	$0.52 \pm 0.19$
$|h_0^{(1)}| \cdot 10^4$	$0.30 \pm 0.23$	$0.30 \pm 0.22$	$0.30 \pm 0.23$	$0.45 \pm 0.26$	$0.32 \pm 0.24$	$0.28 \pm 0.22$
$|h_+^{(1)}| \cdot 10^4$	$0.22 \pm 0.20$	$0.22 \pm 0.19$	$0.22 \pm 0.19$	$0.21 \pm 0.19$	$0.26 \pm 0.22$	$0.22 \pm 0.19$
$|h_-^{(1)}| \cdot 10^4$	$0.23 \pm 0.19$	$0.23 \pm 0.19$	$0.23 \pm 0.20$	$0.30 \pm 0.21$	$0.32 \pm 0.22$	$0.23 \pm 0.19$
$|h_+^{(2)}| \cdot 10^4$	$0.052 \pm 0.045$	$0.053 \pm 0.045$	$0.052 \pm 0.044$	$0.046 \pm 0.042$	$0.064 \pm 0.053$	$0.050 \pm 0.044$
$|h_-^{(2)}| \cdot 10^4$	$0.046 \pm 0.038$	$0.046 \pm 0.039$	$0.046 \pm 0.039$	$0.092 \pm 0.050$	$0.070 \pm 0.047$	$0.045 \pm 0.038$

**Table 3:** Results from the fit for WCs and hadronic contributions in the PDD approach. See Sect. 2.1 for details of the six NP scenarios

Par.	(I)	(II)	(III)	(IV)	(V)	(VI)
$C_{7}^\mathrm{NP}$	−	−	$0.013 \pm 0.014$	$0.008 \pm 0.014$	$0.011 \pm 0.014$	$0.014 \pm 0.014$
$C_{9,\mu }^\mathrm{NP}$	$-1.47 \pm 0.63$	$-1.17 \pm 0.46$	$-1.58 \pm 0.64$	−	$-0.43 \pm 0.23$	$-1.43 \pm 0.64$
$C_{9,e}^\mathrm{NP}$	$0.007 \pm 0.620$	−	$-0.08 \pm 0.63$	−	$0.21 \pm 0.29$	$-1.2 \pm 1.2$
$C_{10,\mu }^\mathrm{NP}$	−	$0.26 \pm 0.23$	−	$0.27 \pm 0.26$	$0.43 \pm 0.23$	$0.20 \pm 0.25$
$C_{10,e}^\mathrm{NP}$	−	−	−	$-0.86 \pm 0.4$	$-0.21 \pm 0.29$	$-0.60 \pm 0.99$
$|h_0^{(0)}| \cdot 10^4$	$2.6 \pm 1.6$	$2.3 \pm 1.4$	$2.6 \pm 1.6$	$1.7 \pm 1.3$	$1.7 \pm 1.3$	$2.6 \pm 1.6$
$|h_+^{(0)}| \cdot 10^4$	$0.075 \pm 0.066$	$0.081 \pm 0.070$	$0.077 \pm 0.067$	$0.086 \pm 0.075$	$0.087 \pm 0.075$	$0.077 \pm 0.067$
$|h_-^{(0)}| \cdot 10^4$	$0.52 \pm 0.21$	$0.55 \pm 0.22$	$0.52 \pm 0.21$	$0.60 \pm 0.23$	$0.59 \pm 0.23$	$0.53 \pm 0.21$
$|h_0^{(1)}| \cdot 10^4$	$0.40 \pm 0.32$	$0.41 \pm 0.34$	$0.39 \pm 0.32$	$0.50 \pm 0.36$	$0.46 \pm 0.37$	$0.40 \pm 0.33$
$|h_+^{(1)}| \cdot 10^4$	$0.40 \pm 0.29$	$0.42 \pm 0.30$	$0.40 \pm 0.29$	$0.39 \pm 0.29$	$0.42 \pm 0.30$	$0.41 \pm 0.30$
$|h_-^{(1)}| \cdot 10^4$	$0.47 \pm 0.35$	$0.52 \pm 0.38$	$0.48 \pm 0.36$	$0.82 \pm 0.46$	$0.73 \pm 0.43$	$0.50 \pm 0.37$
$|h_+^{(2)}| \cdot 10^4$	$0.138 \pm 0.087$	$0.160 \pm 0.099$	$0.131 \pm 0.086$	$0.139 \pm 0.094$	$0.160 \pm 0.100$	$0.145 \pm 0.095$
$|h_-^{(2)}| \cdot 10^4$	$0.112 \pm 0.085$	$0.126 \pm 0.098$	$0.111 \pm 0.083$	$0.190 \pm 0.100$	$0.170 \pm 0.110$	$0.124 \pm 0.094$

**Table 4:** Experimental results (with symmetrised errors) and results from the fit for key observables in the PMD approach. See Sect. 2.1 for details of the six NP scenarios

Obs.	Exp. value	(I)	(II)	(III)	(IV)	(V)	(VI)
${R_K}_{[1,6]}$	$0.753 \pm 0.090$	$0.722 \pm 0.067$	$0.703 \pm 0.047$	$0.722 \pm 0.067$	$0.781 \pm 0.055$	$0.740 \pm 0.061$	$0.724 \pm 0.067$
$$ {R_{K^*}}_{[0.045,1.1]}$$	$$0.680 \pm 0.093$$	$$0.885 \pm 0.016$$	$$0.881 \pm 0.016$$	$$0.885 \pm 0.016$$	$$0.839 \pm 0.024$$	$$0.858 \pm 0.019$$	$$0.843 \pm 0.030$$
$$ {R_{K^*}}_{[1.1,6]}$$	$$0.707 \pm 0.102$$	$$0.803 \pm 0.057$$	$$0.786 \pm 0.049$$	$$0.802 \pm 0.057$$	$$0.713 \pm 0.065$$	$$0.740 \pm 0.060$$	$$0.717 \pm 0.067$$
${R_{K^*}^L}_{[1.1,6]}$	−	$0.724 \pm 0.067$	$0.705 \pm 0.049$	$0.723 \pm 0.067$	$0.722 \pm 0.065$	$0.722 \pm 0.063$	$0.723 \pm 0.068$
$$ {R_{K^*}^T}_{[1.1,6]}$$	−	$$1.053 \pm 0.030$$	$$1.046 \pm 0.069$$	$$1.050 \pm 0.030$$	$$0.692 \pm 0.067$$	$$0.794 \pm 0.053$$	$$0.75 \pm 0.25$$
${R_{\phi }}_{[1.1,6]}$	−	$0.782 \pm 0.060$	$0.764 \pm 0.048$	$0.781 \pm 0.060$	$0.720 \pm 0.064$	$0.737 \pm 0.061$	$0.717 \pm 0.062$
${R_{\phi }^L}_{[1.1,6]}$	−	$0.723 \pm 0.067$	$0.704 \pm 0.049$	$0.722 \pm 0.067$	$0.728 \pm 0.064$	$0.723 \pm 0.063$	$0.721 \pm 0.067$
${R_{\phi }^T}_{[1.1,6]}$	−	$1.044 \pm 0.032$	$1.038 \pm 0.066$	$1.043 \pm 0.033$	$0.691 \pm 0.067$	$0.794 \pm 0.053$	$0.75 \pm 0.24$
${P_5}_{[4,6]}^{\hbox {LHCb}}$	$-0.301 \pm 0.160$	$-0.423 \pm 0.060$	$-0.427 \pm 0.061$	$-0.420 \pm 0.059$	$-0.556 \pm 0.063$	$-0.587 \pm 0.053$	$-0.431 \pm 0.061$
${P_5}_{[6,8]}^{\hbox {LHCb}}$	$-0.505 \pm 0.124$	$-0.602 \pm 0.059$	$-0.607 \pm 0.061$	$-0.598 \pm 0.059$	$-0.677 \pm 0.066$	$-0.704 \pm 0.057$	$-0.61 \pm 0.06$
${P_5}_{[4,6]}^{\hbox {ATLAS}}$	$-0.26 \pm 0.39$	$-0.423 \pm 0.060$	$-0.427 \pm 0.061$	$-0.420 \pm 0.059$	$-0.556 \pm 0.063$	$-0.587 \pm 0.053$	$-0.431 \pm 0.061$
${P_5}_{[4.3,6]}^{\hbox {CMS}}$	$-0.955 \pm 0.268$	$-0.442 \pm 0.059$	$-0.447 \pm 0.061$	$-0.440 \pm 0.059$	$-0.572 \pm 0.063$	$-0.603 \pm 0.053$	$-0.451 \pm 0.061$
${P_5}_{[6,8.68]}^{\hbox {CMS}}$	$-0.660 \pm 0.220$	$-0.623 \pm 0.060$	$-0.626 \pm 0.061$	$-0.618 \pm 0.059$	$-0.688 \pm 0.067$	$-0.711 \pm 0.058$	$-0.63 \pm 0.06$
${P_5}_{[4,8]}^{\hbox {Belle}}$	$-0.025 \pm 0.318$	$-0.520 \pm 0.057$	$-0.524 \pm 0.059$	$-0.516 \pm 0.057$	$-0.620 \pm 0.063$	$-0.649 \pm 0.053$	$-0.528 \pm 0.059$
${P_{5,e}}_{[4,8]}^{\hbox {Belle}}$	$-0.510 \pm 0.272$	$-0.782 \pm 0.054$	$-0.794 \pm 0.039$	$-0.782 \pm 0.054$	$-0.536 \pm 0.063$	$-0.710 \pm 0.044$	$-0.42 \pm 0.23$

**Table 5:** Experimental results (with symmetrised errors) and results from the fit for key observables in the PDD approach. See Sect. 2.1 for details of the six NP scenarios

Obs.	Exp. value	(I)	(II)	(III)	(IV)	(V)	(VI)
${R_K}_{[1,6]}$	$0.753 \pm 0.090$	$0.724 \pm 0.068$	$0.714 \pm 0.064$	$0.723 \pm 0.067$	$0.775 \pm 0.057$	$0.740 \pm 0.062$	$0.719 \pm 0.073$
$$ {R_{K^*}}_{[0.045,1.1]}$$	$$0.680 \pm 0.093$$	$$0.883 \pm 0.017$$	$$0.873 \pm 0.016$$	$$0.883 \pm 0.017$$	$$0.842 \pm 0.024$$	$$0.861 \pm 0.019$$	$$0.847 \pm 0.030$$
$$ {R_{K^*}}_{[1.1,6]}$$	$$0.707 \pm 0.102$$	$$0.803 \pm 0.059$$	$$0.777 \pm 0.053$$	$$0.801 \pm 0.059$$	$$0.714 \pm 0.066$$	$$0.755 \pm 0.059$$	$$0.722 \pm 0.069$$
${R_{K^*}^L}_{[1.1,6]}$	−	$0.726 \pm 0.070$	$0.719 \pm 0.061$	$0.723 \pm 0.070$	$0.726 \pm 0.065$	$0.729 \pm 0.063$	$0.719 \pm 0.073$
$$ {R_{K^*}^T}_{[1.1,6]}$$	−	$$1.050 \pm 0.033$$	$$0.959 \pm 0.083$$	$$1.046 \pm 0.034$$	$$0.688 \pm 0.069$$	$$0.824 \pm 0.049$$	$$0.8 \pm 0.32$$
${R_{\phi }}_{[1.1,6]}$	−	$0.782 \pm 0.062$	$0.761 \pm 0.054$	$0.779 \pm 0.062$	$0.722 \pm 0.065$	$0.749 \pm 0.060$	$0.718 \pm 0.063$
${R_{\phi }^L}_{[1.1,6]}$	−	$0.724 \pm 0.070$	$0.716 \pm 0.061$	$0.721 \pm 0.070$	$0.731 \pm 0.064$	$0.728 \pm 0.063$	$0.716 \pm 0.071$
${R_{\phi }^T}_{[1.1,6]}$	−	$1.039 \pm 0.036$	$0.955 \pm 0.079$	$1.036 \pm 0.036$	$0.693 \pm 0.068$	$0.828 \pm 0.049$	$0.79 \pm 0.3$
${P_5}_{[4,6]}^{\hbox {LHCb}}$	$-0.301 \pm 0.160$	$-0.398 \pm 0.066$	$-0.405 \pm 0.068$	$-0.401 \pm 0.066$	$-0.435 \pm 0.065$	$-0.421 \pm 0.068$	$-0.417 \pm 0.068$
${P_5}_{[6,8]}^{\hbox {LHCb}}$	$-0.505 \pm 0.124$	$-0.536 \pm 0.080$	$-0.521 \pm 0.083$	$-0.539 \pm 0.080$	$-0.521 \pm 0.080$	$-0.508 \pm 0.082$	$-0.545 \pm 0.083$
${P_5}_{[4,6]}^{\hbox {ATLAS}}$	$-0.26 \pm 0.39$	$-0.398 \pm 0.066$	$-0.405 \pm 0.068$	$-0.401 \pm 0.066$	$-0.435 \pm 0.065$	$-0.421 \pm 0.068$	$-0.417 \pm 0.068$
${P_5}_{[4.3,6]}^{\hbox {CMS}}$	$-0.955 \pm 0.268$	$-0.414 \pm 0.067$	$-0.420 \pm 0.068$	$-0.417 \pm 0.067$	$-0.447 \pm 0.066$	$-0.434 \pm 0.068$	$-0.433 \pm 0.068$
${P_5}_{[6,8.68]}^{\hbox {CMS}}$	$-0.660 \pm 0.220$	$-0.550 \pm 0.083$	$-0.531 \pm 0.086$	$-0.553 \pm 0.083$	$-0.529 \pm 0.084$	$-0.514 \pm 0.085$	$-0.556 \pm 0.086$
${P_5}_{[4,8]}^{\hbox {Belle}}$	$-0.025 \pm 0.318$	$-0.472 \pm 0.070$	$-0.467 \pm 0.072$	$-0.475 \pm 0.070$	$-0.481 \pm 0.070$	$-0.467 \pm 0.072$	$-0.486 \pm 0.072$
${P_{5,e}}_{[4,8]}^{\hbox {Belle}}$	$-0.510 \pm 0.272$	$-0.725 \pm 0.075$	$-0.664 \pm 0.095$	$-0.733 \pm 0.075$	$-0.424 \pm 0.063$	$-0.570 \pm 0.066$	$-0.41 \pm 0.22$

## Footnotes

1 For all CMS data we use the 7, 8 TeV combined results, which can be found in https://twiki.cern.ch/twiki/bin/view/CMSPublic/PhysicsResultsBPH13010 .

2 Belle measures the $B^0 \rightarrow K^{*0} \mu \mu$ and $B^+ \rightarrow K^{*+} \mu \mu$ channels together, without providing the mixture ratio. On the theoretical side, we can therefore use these measurements under the approximation that QCD power corrections differentiating the amplitudes of the two channels are small. We have numerically checked that the impact of known QCD power corrections [91] is indeed at the percent level in the observables of interest.

3 This choice is motivated in Ref. [24].
